# Comparative Analysis of Chloroplast Genome of *Meconopsis* (Papaveraceae) Provides Insights into Their Genomic Evolution and Adaptation to High Elevation

**DOI:** 10.3390/ijms25042193

**Published:** 2024-02-12

**Authors:** Shuqi Zhao, Xiaoman Gao, Xiaolei Yu, Tao Yuan, Guiyu Zhang, Chenlai Liu, Xinzhong Li, Pei Wei, Xiaoyan Li, Xing Liu

**Affiliations:** 1 State Key Laboratory of Hybrid Rice, College of Life Sciences, Wuhan University, Wuhan 430072, China; zsq21789@163.com (S.Z.); yuxiaolei127@163.com (X.Y.); 2022202040001@whu.edu.cn (G.Z.); chenlailiu87@163.com (C.L.); imweipei@foxmail.com (P.W.); 2 Key Laboratory of Biodiversity and Environment on the Qinghai-Tibet Plateau of Ministry of Education, College of Life Sciences, Wuhan University, Wuhan 430072, China; 3 Laboratory of Extreme Environment Biological Resources and Adaptive Evolution, School of Ecology and Environment, Tibet University, Lhasa 850000, China; xiaoman_gao@163.com (X.G.); yuantaosw@163.com (T.Y.); lixinzhong2012@163.com (X.L.); 4 Biology Experimental Teaching Center, School of Life Science, Wuhan University, Wuhan 430072, China; xiaoyanlixy@sina.com

**Keywords:** chloroplast genome, *Meconopsis*, high variation region, high altitude adaptation

## Abstract

The *Meconopsis* species are widely distributed in the Qinghai-Tibet Plateau, Himalayas, and Hengduan Mountains in China, and have high medicinal and ornamental value. The high diversity of plant morphology in this genus poses significant challenges for species identification, given their propensity for highland dwelling, which makes it a question worth exploring how they cope with the harsh surroundings. In this study, we recently generated chloroplast (cp) genomes of two *Meconopsis* species, *Meconopsis paniculata* (*M. paniculata*) and *M. pinnatifolia*, and compared them with those of ten *Meconopsis* cp genomes to comprehend cp genomic features, their phylogenetic relationships, and what part they might play in plateau adaptation. These cp genomes shared a great deal of similarities in terms of genome size, structure, gene content, GC content, and codon usage patterns. The cp genomes were between 151,864 bp and 154,997 bp in length, and contain 133 predictive genes. Through sequence divergence analysis, we identified three highly variable regions (*trnD*-*psbD*, *ccsA*-*ndhD*, and *ycf1* genes), which could be used as potential markers or DNA barcodes for phylogenetic analysis. Between 22 and 38 SSRs and some long repeat sequences were identified from 12 *Meconopsis* species. Our phylogenetic analysis confirmed that 12 species of *Meconopsis* clustered into a monophyletic clade in Papaveraceae, which corroborated their intrageneric relationships. The results indicated that *M. pinnatifolia* and *M. paniculata* are sister species in the phylogenetic tree. In addition, the *atpA* and *ycf2* genes were positively selected in high-altitude species. The functions of these two genes might be involved in adaptation to the extreme environment in the cold and low CO_2_ concentration conditions at the plateau.

## 1. Introduction

Chloroplast, a kind of plastid, is a photosynthetic organelle unique to higher plants and some algae, and it has the function of synthesizing starch, fatty acid, pigment, and protein [1]. Chloroplasts have a complete genetic system, and the independent genetic information within the system is called the chloroplast genomes (cp genomes) [2]. Most cp genomes have a typical quadripartite structure, and their covalently closed double-stranded ring structure is the most typical, which can be divided into regions: A large single copy area (LSC), a small single copy area (SSC), and two isometric inverted regions (IRa and IRb). The size of the cp genomes of higher plants generally ranges from 110 to 160 kb. Usually, there are between 120 and 130 genes with coding functions in the cp genomes, which can be roughly divided into 3 categories related to the photosynthetic system, transcription and translation, and other biosynthesis of amino acids, fatty acids, pigments, and so on [2]. The cp genomes are characterized by sequence conservation, structural simplicity, and uniparental inheritance [3]. The cp genomes are relatively simple and easy to obtain. Compared with the nuclear genome, the genetic information of the cp genomes is independent, providing valuable information in tracing the origin of species, revealing the direction of evolution and genealogical structure, and also a means to differentiate among taxa. With the rapid development of molecular biology, the sequencing technology of the genome has gradually become mature [4], along with the continuous innovation of biotechnology, the research at the genetic level has deepened, and the research on the use of genetic information from the cp genomes has gradually increased.

The genus *Meconopsis* is one of the more diverse genera in the Papaveraceae family, with more than 70 species, and it forms an important part of the ecological diversity of the Tibetan Plateau region [5]. The *Meconopsis* species are famous ornamental plants, known for their large flowers and colorful and beautiful appearance; they rank among the most striking flowers found in alpine plants. *Meconopsis* species have a long clinical history in China and other Asian countries, and their medicinal value is highly valued. Plant extracts from the *Meconopsis* species have been reported to possess the effects of clearing heat and detoxification, reducing swelling and pain, and having antioxidant and anti-tumor effects [6,7,8]. These plants are mainly distributed in the Qinghai-Tibet Plateau (QTP), the Hengduan Mountains, and the Himalayan region at an altitude of 2000~5800 m. Their habitats range from temperate forests to alpine meadows and alpine tundra with rhyolite flats [9,10]. The uplift of the QTP, which occurred during the Late Tertiary and Early Quaternary, dramatically changed the distribution and genetic differentiation of the *Meconopsis* species [9,11]. The uplift of the QTP and the evolution of climate caused ecological habitat changes [12]. After geographical isolation and natural selection, the separation of species is formed, which leads to the formation of new species. Frequent hybridization creates opportunities for new species to form, as pollen and seed dispersal or other factors lead to secondary contact between already isolated populations. Isolation, natural selection, and hybridization jointly drive the diversity of the *Meconopsis* species [13]. However, hybridization has also resulted in morphological (e.g., style length and presence of stylar discs) and karyotypic differences in some *Meconopsis* species, making it difficult to classify them on the basis of macromorphological features such as floral colors alone [14]. Consequently, it is crucial to look for more molecular data to improve species identification, and thus explore and refine the phylogenetic relationships within the genus. In comparison to traditional morphological markers, molecular markers have many advantages, including easy assay, stability regardless of environmental or external factors, and representation throughout entire genomes [15]. Therefore, molecular markers are used as important tools for evaluating genetic diversity among plant species and for plant molecular breeding. In plant species, the cp genome is widely used for phylogenetic analyses and molecular marker development to improve phylogenetic resolution at the interspecific level. A great deal of research has been conducted using molecular markers in the study of the phylogeny of the genus *Meconopsis* [16,17]. Previous studies have complemented previous morphology-based treatments on the phylogenetic relationships of a few *Meconopsis* species through large-scale sampling and the construction of phylogenetic models using molecular markers [5]. Numerous studies have concentrated on the medicinal usefulness and phylogeny of the *Meconopsis* species. However, there are few studies examining the adaptive evolution of the *Meconopsis* species to the distinctive alpine habitats they occupy.

Throughout its dispersal to diverse alpine regions, the *Meconopsis* species have undergone rapid evolutionary radiation, which makes it ideal for studying how alpine regions affect species formation and differentiation, and for understanding how these species are adapted to alpine environments [11]. High-altitude habitats typically exhibit distinct environmental features, including low temperature, intense radiation, and low CO_2_ concentration, which may produce some changes in environmental adaptation genes [18,19]. Evolution by natural selection is a direct response to selection pressure. Phylogenetic methods can utilize protein-coding genes (PCGs) to determine gene evolutionary rates and identify natural selection imprints [20]. The cp genomes are conserved and play an integral role in the photosynthetic process. This provides a valuable opportunity to investigate their adaptive evolution. Many plants’ high-altitude adaptation mechanisms focus on the nuclear genome [21,22,23], while there are fewer studies on the accelerated evolution of cp genomes due to high-altitude adaptation [24,25,26]. Previous studies have confirmed that genes of *atp* (*atpA* and *atpF*), *ndh* (*ndhA*, *ndhF*, and *ndhH*), and *ycf* (*ycf1* and *ycf4*) families in chloroplasts of alpine species tend to exhibit higher evolutionary rates than those of low-altitude species [26]. As most *Meconopsis* species are found in alpine environments, it is possible to investigate the selection that their cp genomes undergo during environmental acclimatization.

In this study, our objective was to gather insights from the chloroplast genome to better understand the genetic characteristics and adaptive evolution of the *Meconopsis* species. For the first time, a cp genome characterization and adaptive analysis of *Meconopsis* was carried out. First, the cp genomes of two species of *M. paniculata* and *M. pinnatifolia* were newly sequenced. Together with 10 cp genomes (*M. racemosa*, *M. henrici*, *M. punicea*, *M. quintuplinervia*, *M. pseudohorridula*, *M. simplicifolia*, *M. betonicifolia*, *M. horridula*, *M. integrifolia,* and *M. bella*) available in GenBank, 12 cp genomes were comparatively analyzed using DNA data from NCBI, including genome size, gene content, structure, GC content, IR boundaries, nucleotide diversity, codon usage, and SSR distribution. On this basis, together with the cp genomes of 44 other Papaveraceae species (all available in NCBI), we reconstructed part of the topology of Papaveraceae and investigated the phylogenetic relationships of 12 species of the genus *Meconopsis* in the Papaveraceae family. In addition, we investigated some genes and loci at the genus level that may have accelerated evolution induced by extreme environments. In summary, in this study, we aimed to (1) understand the characteristics of the cp genomes of *Meconopsis* (two newly collected and all available in NCBI) and its evolutionary process and (2) understand whether natural selection has influenced the cp genomes of *Meconopsis* in adaptation to the extreme high-altitude environment.

## 2. Results and Discussion

### 2.1. Characterization of the CP Genome Structure of Meconopsis Species

**Figure 1:**
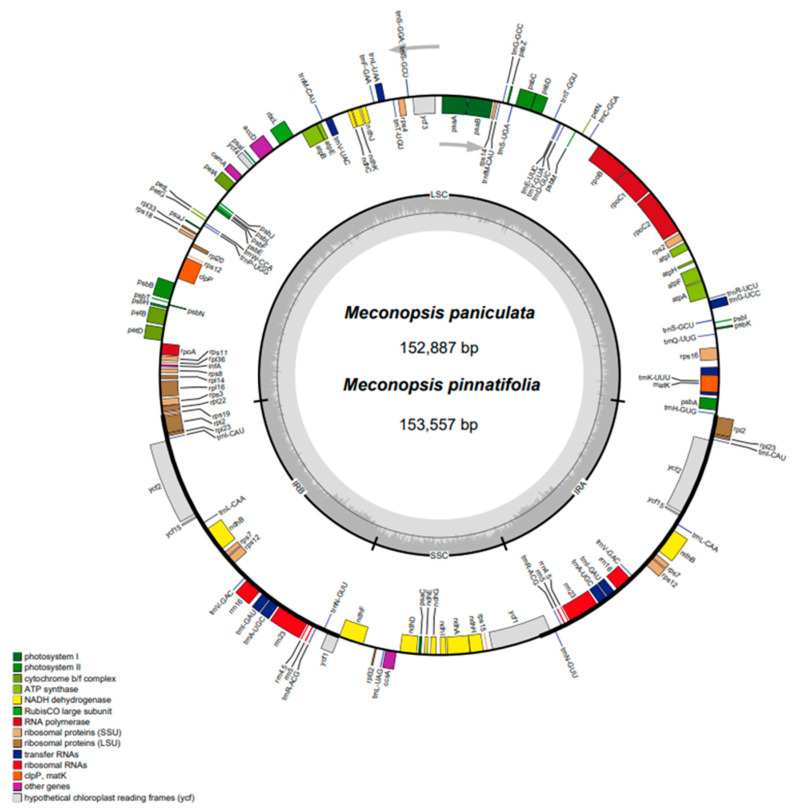
The cp genome map of two newly sequenced *Meconopsis* species, *M. paniculata* and *M. pinnatifolia*. The outer circle showed the transcription direction; genes inside were transcribed in the clockwise direction while genes outside were transcribed counterclockwise. LSC/SSC/IR zones were shown in the inner circle. Genes belonging to different functional groups were color coded.

After filtering, the two newly sequenced species yielded over 4 gigabases (Gb) of clean data. When mapped with clean short reads, both data were assembled into high-quality contigs without any gap. These contigs were subsequently circularized, fully annotated, and manually checked against other cp genomes in the Papaveraceae family (Figure 1; Table 1). Overall, the cp genomes of the 12 species in the genus *Meconopsis* exhibited similar structural features (Table 2).

All 12 cp genomes had a typical 4-part structure, including 2 equidistant IR regions (IRb/IRa, ranging from 25,521~26,178 bp), one LSC region (82,809~85,153 bp) and one SSC region (17,646~17,905 bp). Among them, *M. integrifolia* cp genome size was the smallest (151,864 bp) and *M. quintuplinervia* cp genome size was the largest (154,997 bp). The total GC (guanine-cytosine) content was almost the same (38.5–38.9%), and the GC content of the two IR regions (42.9–43.2%) was higher than that of the LSC region (37.0–37.5%) and the SSC region (32.7–33.5%). The cp genomes exhibited a clear AT preference, which was most pronounced in the SSC region. In addition, all cp genomes had highly similar gene contents, consisting of 88 unique PCGs, 37 unique tRNA genes, and 8 unique rRNA genes, with 7 or 8 PCGs, 4 rRNA genes, and 7 tRNA genes located in the IR region being replicated (Table 1 and Table 2). The second copies of the *rps19* and *ycf1* genes of the cp genome of the genus *Meconopsis* were in a pseudogenized state. In all cp genomes, 18 PCGs and tRNA genes were detected to contain introns, with 2 PCGs, *ClpP*, and *ycf3*, containing 2 introns (Table 1). There were no significant differences in genome size, GC content, gene order, and gene content compared to cp genomes of other genera in the Papaveraceae family, which is consistent with observations in other higher plants [27,28].

### 2.2. IR Boundary Analysis

**Figure 2:**
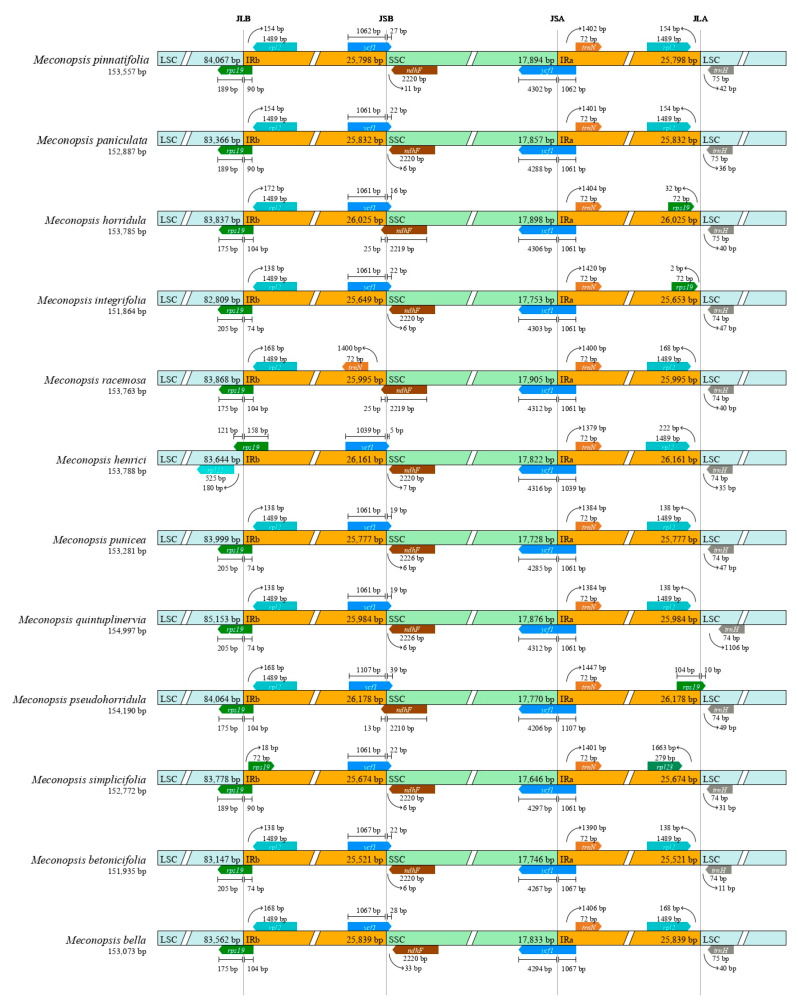
The comparison of the LSC, IR, and SSC border regions among the 12 *Meconopsis* chloroplast genomes. JLB, JSB, JSA, and JLA denoted the junction sites of LSC and IRb, IRb and SSC, SSC and IRa, and IRa and LSC, respectively. The number above the gene features refers to the distance between the ends of genes and the border sites.

Genome structure, including gene number and gene order, is highly conserved in the *Meconopsis* species. However, structural changes still exist at the LSC/IR/SSC boundary. The contraction and expansion of the IR/SC boundary within the plant cp genome determines its size and are the primary mechanisms driving genome-wide size variations [29]. IR/SC boundary changes have been widely documented as a common evolutionary phenomenon reflecting cp genome expansion and contraction, leading to gene pseudogenization near the boundary [30]. We selected *M. pinnatifolia* as a control and investigated the boundaries of IR, LSC, and SSC regions to compare the cp genome structure. We investigated the boundaries of IR, LSC, and SSC regions in the *Meconopsis* species, Figure 2 showed some variations, and upon comparison, the boundaries did not exhibit significant differences. In most *Meconopsis* species, the *ndhF* gene was located predominantly in the SSC region, with its right end extending into the IRb region. In the case of most *Meconopsis* species, the *ycf1* gene was situated on both sides of the SSC region and the IRa region, with a length ranging between 5312 and 5372 bp. Due to its specific location, another copy of *ycf1* was found at the border of the IRb and SSC regions, resulting in a truncated pseudogene. The *rps19* genes were positioned at the junction of the LSC and IRb regions. Similar to *ycf1*, they were also found at the junction of IRa, and in most instances, the second copy at the junction of IRa and LSC was also in a pseudogenized state. In contrast, in *M. simplicifolia*, the *rps19* gene was situated at the junction of LSC and IRb, while the second copy was located within IRb. The *rps19* genes in these species had evidently lost their protein-coding capacity because they were partially replicated in the IRb region, resulting in a pseudogene for *rps19*, mirroring the situation observed for the *ycf1* gene.

### 2.3. Genomic Sequence Divergence

Plant DNA barcoding refers to the use of gene sequencing technology to analyze and compare a segment of DNA sequence (approximately a chloroplast DNA fragment of about 650 bp) within a plant to determine the identity of a species by comparing the differences in DNA sequences between different species. DNA barcoding has become a fast and reliable molecular tool in species formation and taxonomy [31,32]. Previous studies showed that *rbcL*, *matK*, *trnH-psbA* in the cp genome and ITS in the nuclear sequence were universal DNA barcodes for land plants due to their high specificity and amplification efficiency [33]. However, due to the short DNA barcode sequences and limited genetic information, they are not specific enough to distinguish closely related species [34]. Therefore, there is a need to combine them with other molecular markers to improve the efficiency and accuracy of DNA barcoding technology for precise identification.

**Figure 3:**
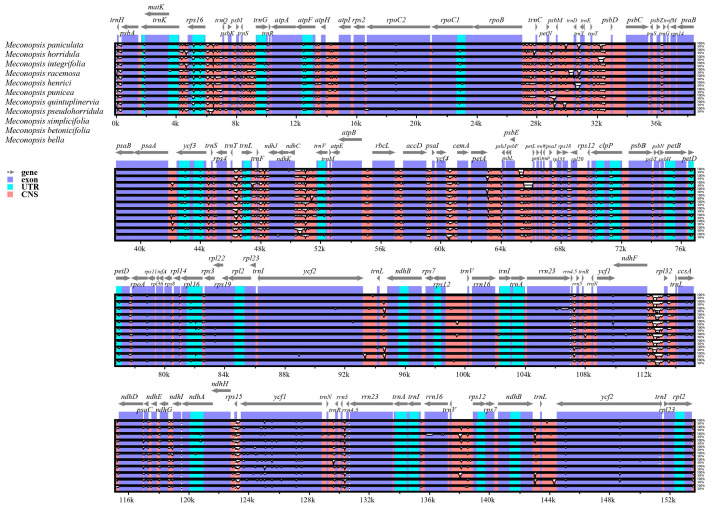
Sequence identity plot comparing the 12 *Meconopsis* chloroplast genomes with *M. pinnatifolia* as a reference by using mVISTA. The horizontal axis represents the coordinates of cp genomes in the alignment result. Exons, introns, and conserved noncoding sequences (CNSs) are marked as different colors.

First, mVISTA is a bioinformatic tool that allows global or multiple sequence comparisons of chloroplast or mitochondrial genomes, revealing similarity and rearrangement information. The cp genome of *M. pinnatifolia* was used as a reference for comparison with 11 other cp genomes (Figure 3). As expected, most of the PCGs had high concordance. In non-coding regions, such as the intergenic regions *trnD-trnY*, *trnT-psbD*, *petA-psbJ*, *psbE-petL*, and *ccsA-ndhD*, significant variations were observed. Within the coding region, the *ycf1* gene interval showed significant variation. Notably, *M. paniculata*, *M. integrifolia*, and *M. henrici* showed more and greater variation than other *Meconopsis* species.

**Figure 4:**
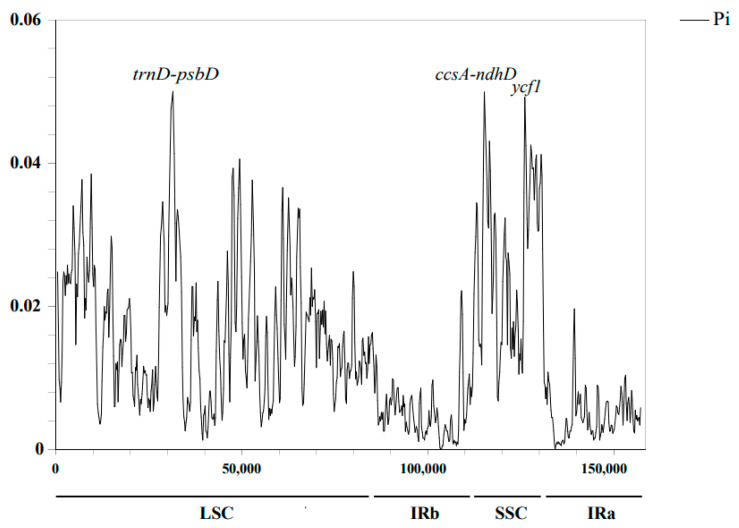
Comparative analysis of the nucleotide polymorphism (Pi) values among the 12 cp genomes of *Meconopsis*.

The Pi values of these *Meconopsis* cp genomes were then calculated to validate the visualization results obtained from mVISTA and to further detect highly variable regions. As shown in Figure 4, both results indicated that the IR region was much more conserved than the LSC and SSC regions. The Pi values for the entire cp genome ranged from 0 to 0.06 when analyzed for all 12 species (Figure 4; Table S2). Six intergenic regions (*trnD-psbD* and *ccsA-ndhD*) and part of the *ycf1* gene were more variable than the others (0.45–0.06), which was in line with the results of the mVISTA analysis. Among these regions, *trnD-psbD* exhibited the highest variability with a Pi value of 0.05.

Combining the results of Pi calculations with the mVISTA findings described above, we found that the entire cp genome exhibited regular features related to the phylogenetic relationships among the 12 species of the genus *Meconopsis*. Notably, three species within the genus *Meconopsis*, namely *M. paniculata*, *M. integrifolia*, and *M. henrici*, displayed alterations in regions of high variation. This observation suggests that there might be specific variations in different segments of the genus and that the search for highly variable loci within these changing segments could potentially enhance the precision of species identification.

### 2.4. Codon Usage Analysis

**Figure 5:**
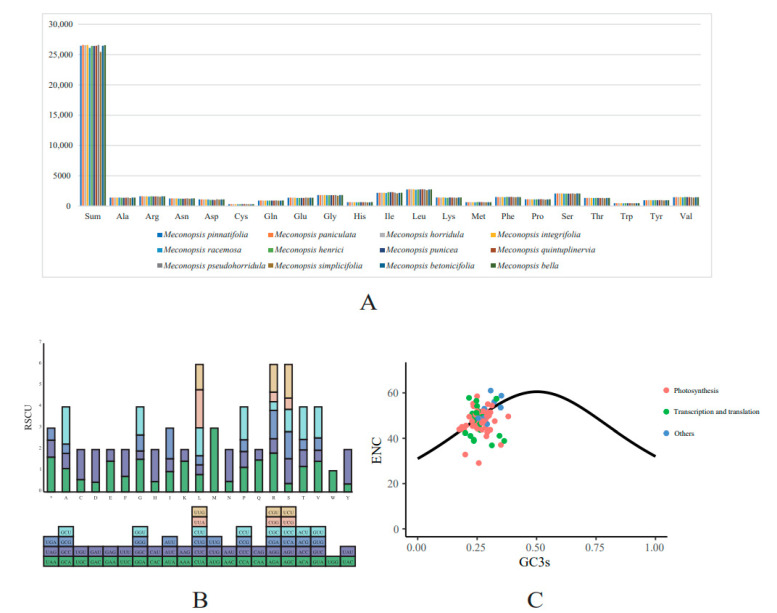
Usage preference of amino acids (AAs) and codons for PCGs. (**A**) AA usage of all the PCGs in each *Meconopsis* species. (**B**) RSCU for every AA in *M. pinnatifolia*. For each amino acid, a color represented a unique codon. (**C**) ENC-GC3 plot for *M. pinnatifolia*; each gene was displayed as a dot, and different colors mean genes in distinct functional groups.

Codon usage bias is an important factor reflecting the evolution of the cp genome. In general, factors such as mutation, natural selection, and phylogenetic relationships may lead to differences in codon use preferences [35]. We analyzed codon usage bias and relative synonymous codon usage (RSCU) of the shared PCGs of 12 *Meconopsis* species in this work. The genus *Meconopsis* had highly similar codon usage preferences and amino acid frequencies (Figure 5A,B and Figure S1; Table S3). In addition to the termination codons, we identified a total of 25,454 ~ 26,597 codons. Leu (10.36–10.49%), isoleucine (8.27–8.72%) and serine (7.77–7.92%) were used more frequently, and cysteine (1.17–1.26%), tryptophan (1.77–1.81%), methionine (2.41–2.56%), and histidine (2.41–2.49%) were used less frequently. Due to the simplicity of codons, most amino acids have multiple synonymous codons; for example, isoleucine has four codons. Nevertheless, it is important to note that only tryptophan and methionine do not have alternative codons [36]. Similar to other higher plants, for plants using multiple codons, the third nucleotide of the codon was more frequently occupied by A/T than C/G [37,38].

ENC-plot analysis was performed on each PCG. The results showed that the PCGs of the *Meconopsis* species had a consistent codon bias pattern (Figure 5C and Figure S2; Table S4). The calculated ENCs of most genes ranged from 30 to 60. Most of the PCGs were located near the expected ENC, suggesting that these genes were mainly random mutations. The distributions of a few photosynthesis-related genes and translation-associated ribosomal proteins were well below the standard curve, suggesting that natural selection or other factors might play an important role in shaping the evolution of these genes.

### 2.5. Repeat Sequence Analysis

**Figure 6:**
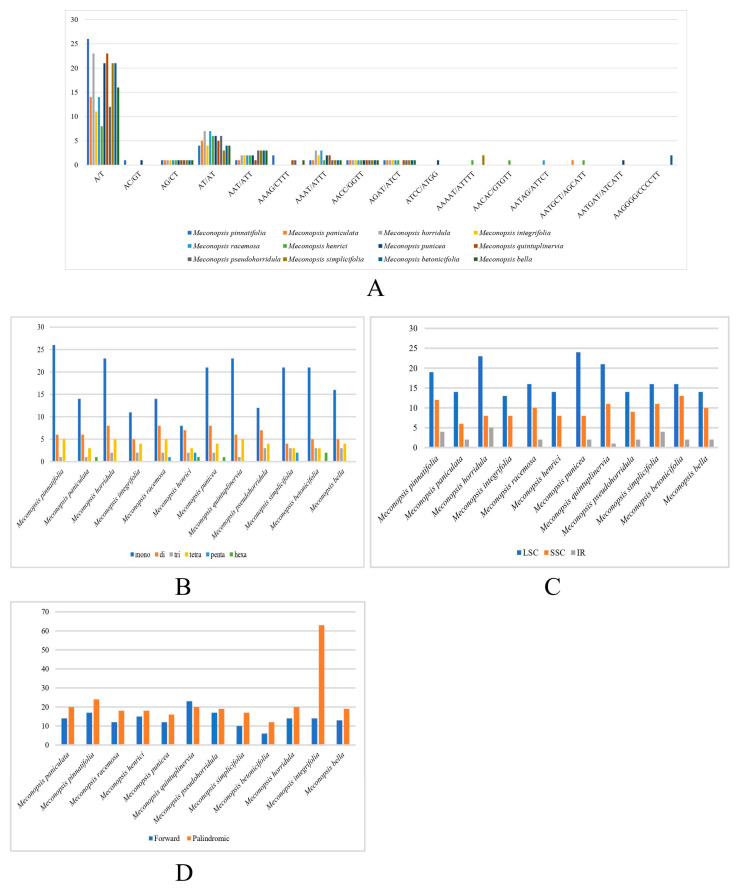
Repeats analysis among cp genomes of *Meconopsis*. (**A**) Distribution of all repeat units for SSRs in each species. (**B**) The number of different types of SSRs in each species. (**C**) Distribution of SSRs, respectively, in LSC, SSC, and IR regions. (**D**) The number of different types for long repeats.

SSRs have been described as powerful tools for species identification, population genetics, and phylogenetic studies [39,40,41]. The study identified 22 to 38 SSRs in 12 *Meconopsis* species (Figure 6B; Table S5). The highest content of SSRs was found in *M. pinnatifolia* and *M. horridula*. Among these repeat sequences, single-nucleotide SSRs were the most abundant (8–26) and consisted mainly of A/T. Some regular repeat sequences, such as A/T, AG/CT/AT, and AAAT/ATTTT/AACC/GGTT, were shared across all cp genomes, whereas other repetitive units of more than four nucleotides, such as ATCC/ATGG, AAAAT/ATTTTT, and AAGGGG/CCCCCTT, were more pronounced in specific cp genomes (Figure 6A). Among all cp genomes, the LSC contained the highest number of SSRs and two IRs contained the lowest number of SSRs, which was consistent with the previously mentioned pattern of Pi analysis (Figure 6C). The length of the repeated sequences in the 12 cp genomes ranged from 10 to 18 bp. Only forward and palindromic repeats were present in all *Meconopsis* cp genomes (Figure 6D; Table S6). A number of large and scattered repeat sequences were believed to be linked to genetic rearrangements and were considered to have a significant role in genome evolution [42,43]. In general, these repetitive sequences could prove invaluable for future population genetics studies.

### 2.6. Phylogenetic Analysis

**Figure 7:**
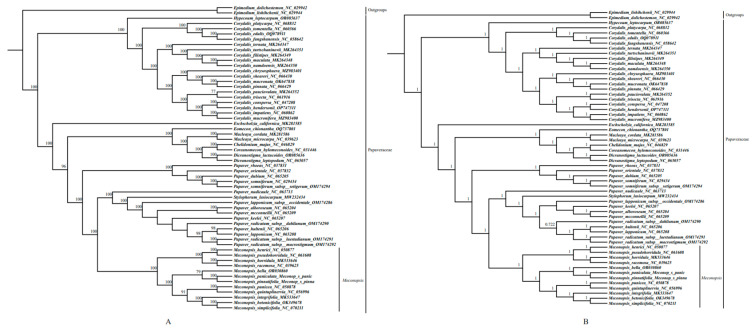
(**A**) Maximum likelihood (ML) phylogenetic tree of 58 species, reconstructed with 132 PCGs (bootstrap below 70% are hidden). (**B**) Bayesian Inference (BI) phylogenetic tree of 58 species, reconstructed with 132 PCGs (bootstrap below 0.7 are hidden). Two non-Papaveraceae species, *Epimedium dolichostemon*, and *Epimedium lishihchenii*, were set as outgroups.

In order to investigate the phylogenetic relationships of the 12 *Meconopsis* species and the phylogenetic position of the genus *Meconopsis* within the Papaveraceae family, a phylogenetic tree was reconstructed using ML and BI methods using a total of 132 PCGs shared in the cp genomes of the 58 plants. The 56 species of 58 plants mentioned above belonged to the Papaveraceae family, and the outgroups were 2 non-Papaveraceae species, *Epimedium dolichostemon* (Berberidaceae) and *Epimedium lishihchenii* (Berberidaceae). The topologies generated by both methods were almost identical except for the Papaver genus and all nodes were well supported by high ML bootstrap and Bayesian posterior probability (Figure 7). Because of the high morphological and ecological diversity of the genus *Meconopsis*, the classification of the plants in the genus *Meconopsis* was relatively complex at the intra-generic level. As can be seen in Figure 7, these *Meconopsis* species could be divided mainly into four major clades, of which *M. punicea*, *M. quintuplinervia*, *M. integrifolia*, *M. betonicifolia*, and *M. simplicifolia* belong to *subgen. Grandes*, and *M. henrici*, *M. pseudohorridula*, *M.racemosa* and *M. horridula* belong to *subgen. Cumminsia*. The result was broadly similar to those of previous phylogenetic studies [5]. Phylogenetic analyses based on cp genomes showed that *M. pinnatifolia* and *M. paniculata* are sister species. In contrast to previous studies, the *Meconopsis* species were consistent in subgeneric classification but differed in sectional classification. Although *M. betonicifolia* and *M. simplicifolia* are sister species in the phylogenetic tree and both belong to *subgen. Grandes*, *M. betonicifolia* belongs to *sect. Grandes*, while *M. simplicifolia* belongs to *sect. Simplicifoliae* [5]. The use of different molecular markers and an increase in the variety of species studied might lead to changes in some branches of the phylogenetic tree, so we need to develop DNA barcodes with higher resolution to optimize the results and get more precise phylogenetic relationships.

### 2.7. Selection and Adaptation Analyses

**Figure 8:**
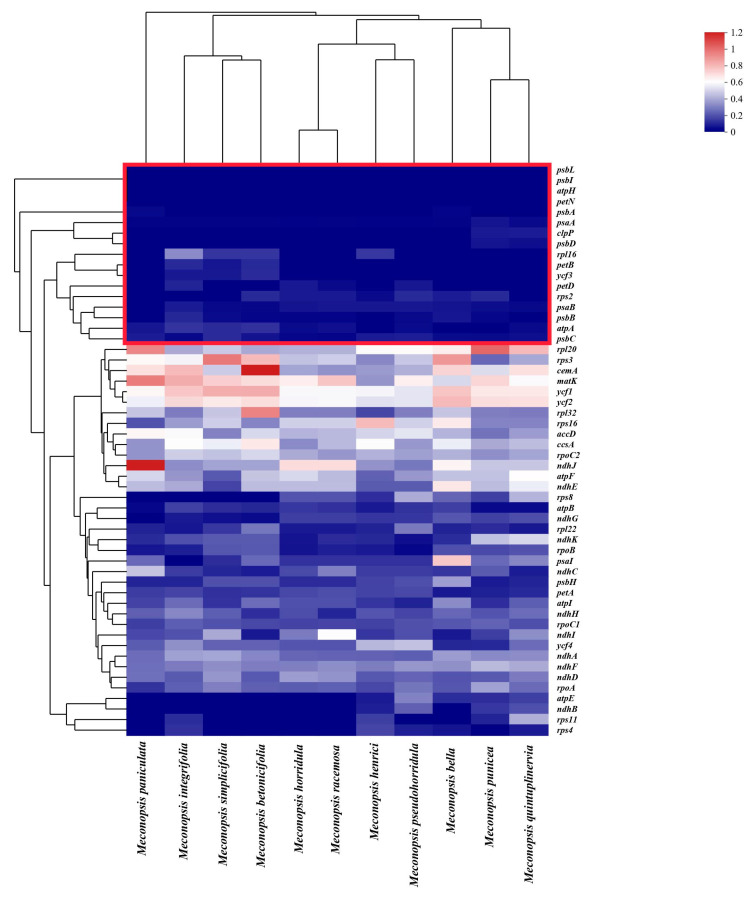
Heatmap representing pairwise Ka/Ks ratios of PCGs among the *Meconopsis* species. The color bias toward red indicates that there is a higher Ka/Ks ratio between genes.

First, we calculated Ka/Ks ratios for the 88 unique PCGs between any 2 *Meconopsis* species, using *M. pinnatifolia* as a reference. Most of the genes were in a state of purifying selection (Ka/Ks ratio < 1) and most of them were in the range of 0~0.4, indicating that most of the PCGs were very conserved in the *Meconopsis* cp genomes at the amino acid level (Table S7). As can be seen from the heatmap, most of the conserved genes within the red line boxes were photosynthesis-related genes (Figure 8). Only a few genes, such as the *cemA* gene in the comparison of *M. betonicifolia* and *M. pinnatifolia* and the *ndhJ* gene in the comparison of *M. paniculata* and *M. pinnatifolia* were under accelerated selection (Ka/Ks ratio > 1).

Natural selection pressures among the *Meconopsis* species were detected using the 88 unique PCGs. Compared with other *Meconopsis* species distributed mainly at low altitudes, *M. racemosa*, *M. henrici*, *M. pseudohorridula*, and *M. horridula* grow at relatively high altitudes with colder climates and higher light radiation [44]. Therefore, whether species surviving in harsh environments at high altitudes have undergone adaptive evolution is a question worth investigating. Four species (*M. racemosa*, *M. henrici*, *M. pseudohorridula*, and *M. horridula*) living in relatively high-altitude ecological niches were used as foreground branches, and selection pressures were estimated using the branch-site model. The results showed that genes *atpA* and *ycf2* were under positive selection (Table S7).

The *atp* and *ycf* families are frequently reported to be involved in adaptation to highland environments [45,46] and are now also confirmed in the *Meconopsis* species in this study. The *atpA* gene is a photosynthesis-related cp gene involved in energy metabolism that is relatively evolutionarily conserved and encodes the ATP synthase CF1 α-subunit protein. The ATP synthase CF1 α-subunit protein is a key enzyme in energy metabolism in plants and plays an important role in a variety of cellular processes. Studies have shown that ATP synthase activity is strongly associated with low-temperature stress. Under low-temperature stress, ATP synthase activity decreases, and ATP content is reduced. ATP synthase activity recovers upon restoration of warmth, and it is critical for plant response to environmental stresses (e.g., plant cold tolerance) [47]. Considering the accelerated evolution of the *atpA* gene under natural selection at high altitude, low temperature, and low CO_2_ in the QTP, it may promote ATP synthase specialization and increase the efficiency of energy transduction for photosynthesis, and therefore, may be involved in the adaptive response of the *Meconopsis* species to environmental stresses.

The *ycf2* gene is the largest plastid gene reported in angiosperms. The *ycf2* gene’s function is largely unknown but does not appear to be specifically related to photosynthesis [48]. The *ycf2* gene has also been shown to encode a protein that is part of the ycf2-FtsHi Heteromeric AAA-ATPase Complex, which is closely related to the TIC complex and plays a role in chloroplast inner membrane where it plays a role in preprotein translocation [49]. The *ycf2* gene, although it varies considerably across cp genomes, acts as a variable gene in many plant cp genomes and is involved in many biological functions [50,51]. Although no study has shown that the *ycf2* gene is associated with adaptation to highland environments, the positive selection of the *ycf2* gene suggests that the *ycf2* gene may have other functions to help plants adapt to extreme environments, which are worth exploring in depth.

## 3. Materials and Methods

### 3.1. Plant Material Sampling, DNA Extracting, and DNA Sequencing

In this study, we investigated a total of 12 *Meconopsis* species, with special attention given to 2 specimens newly acquired from the QTP region of China in 2022. These specimens were collected at altitudes of 3632 m (*M. paniculata*) and 3944 m (*M. pinnatifolia*), as detailed in Table S1. The identification of these specimens was carried out by Professor Xing Liu from Wuhan University in China. To ensure the accessibility of these specimens, voucher samples were preserved in the herbarium of Wuhan University. In the process of gathering plant material, we prioritized the collection of young, fresh, and healthy leaves, which were promptly flash-frozen in liquid nitrogen. This preservation was followed by storage at −80 °C. Subsequently, we implemented a modified CTAB method to extract high-quality genomic DNA. The quality and concentration of the DNA were assessed by agarose gel electrophoresis. If a clear band with a large molecular weight appeared, the quality and concentration of the DNA were proved to be good. If electrophoretic tailing occurred, the DNA was degraded. The next step involved the construction of libraries, wherein genomic DNA underwent fragmentation and adapter ligation. For sequencing, we employed the Illumina Novaseq 6000 platform. This process included additional steps for amplification and purification. All the read data generated throughout this study have been documented and deposited in the NCBI Sequence Read Archive (SRA). Access to this dataset is available under the respective accession numbers SRR25926012 and SRR25926011, specific to each specimen.

### 3.2. Chloroplast Genome De Novo Assembly and Annotation

We utilized the Fastp v0.19.4 software to filter the raw data, which involved the elimination of adapter sequences as well as the removal of low-quality reads [52]. For the de novo assembly of the cp genomes of *M. paniculata* and *M. pinnatifolia*, we employed GetOrganelle v1.7.0 [53]. Subsequently, the assembled contig underwent correction using Pilon and validation through short-read mapping to the contig using Bowtie2 2.3.2 [54,55]. We used Geneious 8.0.4 to visualize the results. The contig was comprehensively covered by clean data through the alignment process [56]. Next, Geseq annotated the well-assembled sequence [57]. tRNAscanSE 2.0.5 software with the default setting was used to identify tRNA genes [58]. After aligning with a set of reference cp genomes, the initial annotations were systematically validated, and adjustments were made to determine the positions of start codons, terminal codons, and introns. Following alignment with a set of reference cp genomes, the original annotations were manually checked and adjusted to ensure their positions for initial codons, terminal codons, and introns were correct. The newly obtained cp genomes were deposited in GenBank with accession numbers OR521090 and OR521089.

### 3.3. Chloroplast Genome Visualization and Sequence Divergence Analysis

The basic features of 12 cp genomes were compared and analyzed using Geneious 8.0.4, including different regions, their GC content, and the proportions of different sequences [56]. In order to visualize the transcriptional direction, position of genes, and the structure feature of each cp genome, the circular maps of *M. paniculata* and *M. pinnatifolia* were drawn by using OrganellarGenomeDRAW (OGDRAW) 1.3.1 [59]. The alignments of the 12 complete cp genome sequences were compared using mVISTA with a LAGAN mode in order to show the variation region of the cp genome among the genus *Meconopsis* [60,61]. The gene distribution at the LSC, SSC, IRa, and IRb boundaries was further revealed using the online tool CPJSdraw [62]. Twelve cp genomes were aligned together. The nucleotide polymorphism (Pi) among cp genomes was calculated, intercompared, and visualized using DnaSP 6.12.03 software, employing a window length of 600 bp and a step size of 200 bp [63].

### 3.4. Analysis of Codon Usage

The relative synonymous codon usage (RSCU) of all the PCGs was calculated using Mega X [64]. RSCU stands for the preference for codon usage, which refers to the relative probability of a codon encoding a corresponding amino acid among synonymous codons for a particular codon. When the RSCU of a codon is greater than 1, it indicates that the codon is preferred when encoding the same amino acid.

The effective number of codons (ENC) is an important index to reflect the degree of non-equilibrium use preference of synonymous codons [65]. In general, the synonymous codon preference of highly expressed genes is larger, so the ENC value is smaller. GC3 refers to the GC content of the third position of all codons in the gene, that is, the frequency of G and C in the third position of the codon, in addition to methionine, tryptophan, and stop codons. CodonW v1.4.4 (JF Peden, Nottingham, UK) was used to calculate the GC3s and ENC of each PCG among the cp genomes. After comparing the results, the standard curve was constructed and shown using an R script. The standard curve in the ENC-GC3 graphic illustrated how the ENC-GC3 content formula fitted. The predominant cause of the observed codon bias stemmed from a variance in nucleotide composition at the third position of the codon, a phenomenon largely driven by mutation. This was particularly evident when the calculated effective number of codons (ENC) for a gene closely approximated the values outlined in the standard curve. Conversely, the position of a gene far below the standard curve indicated that the codon preference of the gene was affected by natural selection and other factors [66].

### 3.5. Analysis of Repeat Sequences in Organelle Genomes

The positions and types of simple sequence repeats (SSRs) in cp genomes were ascertained using MISA [67]. The minimum numbers of repeats were 10, 5, 4, 3, 3, and 3 for mono-, di-, tri-, tetra-, penta-, and hexanucleotides, respectively. REPuter was used to determine the positions and types of simple sequence repeats, employing specific parameters: A hamming distance of 3 and a minimum repeat size of 30 bp [68].

### 3.6. Phylogenetic Analysis

In order to investigate the phylogenetic relationships of the 12 *Meconopsis* species and the phylogenetic position of genus *Meconopsis* within the Papaveraceae family, we chose 58 species (all species of the Papaveraceae family of which cp genomes are available in NCBI, 2 newly collected *Meconopsis* species and 2 non-Papaveraceae species) to reconstruct a phylogenetic tree. These two non-Papaveraceae species were selected as outgroups because these two species were used as outgroups in the analysis of the chloroplast genomes of *Meconopsis* in previous studies [69]. A total of 132 common PCGs were extracted, aligned, and subsequently combined into a matrix using PhyloSuite v1.2.3 after manual assessment and modification [70]. The phylogenetic tree was then reconstructed using *Epimedium lishihchenii* and *Epimedium dolichostemon* as outgroups. For 8000 rapid bootstraps, ModelFinder automatically picked the TVM model for maximum likelihood (ML) analysis using IQ-TREE [71]. Subsequently, BI phylogenies were conducted with the assistance of MrBayes 3.2.6 and the GTR + I + G + F model (2 parallel runs, 2,000,000 generations), with the initial 25% of the sampled data being discarded as burn-in [72]. The Interactive Tree of Life displayed two created trees [73].

### 3.7. Selective Analysis

To assess the selective pressure on the *Meconopsis* species in high-altitude habitats, we conducted an analysis to determine the Ka/Ks ratio (where Ka represents the nonsynonymous substitution ratio and Ks stands for the synonymous substitution ratio) within a study involving 12 *Meconopsis* species. Using PhyloSuite, 88 common PCGs were extracted individually and simultaneously translated to their amino acid counterparts. By invoking KaKs_calculator 2.0, ParaAT was employed to automatically prepare intermediate files and calculate the Ka/Ks value [74,75,76]. Some rows (*infA*, *petG*, *petL*, *psaC*, *psaJ*, *psbE*, *psbF*, *psbJ*, *psbK*, *psbM*, *psbN*, *psbT*, *psbZ*, *rbcL*, *rpl14*, *rpl23*, *rpl33*, *rpl36*, *rps12*, *rps14*, *rps15*, *rps18*, *rps19*, *rps7*, and *rpl2*) and columns (*M. horridula*–*M. paniculata*) with too much Na were discarded. The extremely low synonymous substitution ratio might be to blame for this. If the Ka/Ks ratio was greater than 1, the gene pair was identified to be under positive selection, while a ratio lower than 1 indicated purifying selection.

The PAML v4.10.6 codeml program’s branch-site model was used to calculate the selection pressure brought on by environmental adaptation in the *Meconopsis* species [20]. The degree of selective pressure was gauged by utilizing the ratio of synonymous substitution rate (dS) to nonsynonymous substitution rate (dN). A likelihood ratio test (LRT) was conducted to compare the alternative model (“model = 2, NSsites = 2, omega = 0.5|1.5, and fix_omega = 0”) and the null model (“model = 2, NSsites = 2, omega = 1, and fix_omega = 1”), with the *p*-value of the LRT being examined by the Chi-squared test. Additionally, amino acid positions that could be subject to positive selection were assessed and selected using the Bayesian empirical Bayes (BEB) method. An amino acid site with a posterior probability greater than 0.95 was considered highly likely to be under positive selection, whilst a gene with a *p*-value of 0.05 and ω > 1 was assumed to be under positive selection.

## 4. Conclusions

In this study, 12 *Meconopsis* species were chosen for comprehensive analyses, including comparative, phylogenetic, and adaptive analyses of *Meconopsis* cp genomes. The results showed that the size, structure, GC content, gene content, and genome components of the cp genomes of all *Meconopsis* species were basically the same, and there was no rearrangement of gene order. In the sequence divergence analysis, we detected 3 highly variable regions (*trnD-psbD*, *ccsA-ndhD*, and *ycf1* genes) in 12 *Meconopsis* species, which provided valuable reference evidence for the more accurate identification of the *Meconopsis* species in subsequent studies. In this section, the IR region was also detected to be more conserved than the SSC and LSC regions. The phylogenetic tree reconstructed using PCGs of the cp genome further demonstrated that the 12 *Meconopsis* species in this study belonged to 4 different sections. Meanwhile, three *Meconopsis* species (*M. henrici*, *M. pseudohorridula*, and *M. horridula*) at relatively high-altitude positions were classified as Subgen. Cumminsia. This phylogenetic relationship was also evidenced by various other characteristics of the cp genome, as shown in the mVISTA analyses variation across the cp genome as well as the Pi values calculated between the different segments. In terms of altitudinal adaptation, at the branching and site level, the *atpA* and *ycf2* genes might be under positive selection for species growing at relatively high altitudes, suggesting the contribution of these two genes to adaptation to extreme environments. The above findings provided insights into the conservation and differentiation of the *Meconopsis* cp genome and laid the foundation for accurate species identification. Of course, larger-scale sampling is needed to learn more about the evolutionary features and environmental adaptation patterns of the *Meconopsis* cp genome.

## Figures and Tables

**Table 1:** Gene annotation of the M. paniculata and M. pinnatifolia chloroplast genome.

Category	Group	Genes
Photosynthesis related genes	Rubisco	*rbcL*
	Photosystem I	*psaA, psaB, psaC, psaI, psaJ*
	Photosystem II	*psbA, psbB, psbT, psbK, psbI, psbH, psbM, psbN, psbD, psbC, psbZ, psbJ, psbL, psbE, psbF*
	ATP synthase	*atpA, atpB, atpE, atpF ^a^, atpH, atpI*
	Cytochrome b/f complex	*petA, petB ^a^, petD ^a^, petN, petL, petG*
	Cytochrome C synthesis	*ccsA*
	NADPH dehydrogenase	*ndhA ^a^, ndhB ^a,c^* (×2), *ndhC, ndhD, ndhE, ndhF, ndhH, ndhG, ndhJ, ndhK, ndhI*
Transcription- and translation-related genes	Transcription	*rpoA, rpoB, rpoC2, rpoC1 ^a^*
	Ribosomal proteins	*rps2, rps3, rps4, rps7 ^c^* (×2)*, rps8, rps11, rps12 ^a,c^* (×2)*, rps14, rps15, rps16 ^a^, rps18, rps19, rpl2 ^a,c^* (×2)*, rpl14, rpl16 ^a^, rpl20, rpl22, rpl23 ^c^* (×2)*, rpl32, rpl33, rpl36*
	Translation initiation factor	*infA*
RNA genes	Ribosomal RNA	*rrn16 ^c^* (×2)*, rrn23 ^c^* (×2), *rrn4.5 ^c^* (×2)*, rrn5 ^c^* (×2)
	Transfer RNA	*trnH-GUG, trnK-UUU ^a^, trnQ-UUG, trnS-GCU, trnS-UGA, trnS-GGA, trnG-GCC ^a^, trnR-UCU, trnR-ACG ^c^* (×2)*, trnC-GCA, trnD-GUC, trnY-GUA, trnE-UUC, trnT-UGU, trnG-UCC, trnfM-CAU, trnL-CAA ^c^* (×2)*, trnL-UAA ^a^, trnL-UAG, trnF-GAA, trnV-GAC ^c^* (×2)*, trnV-UAC ^a^, trnM-CAU, trnT-GGU, trnW-CCA, trnP-UGG, trnI-CAU ^c^* (×2)*, trnI-GAU ^a,c^* (×2)*, trnA-UGC ^a,c^* (×2), *trnN-GUU ^c^* (×2)
Other genes	RNA processing	*matK*
	Carbon metabolism	*cemA*
	Fatty acid synthesis	*accD*
	Proteolysis	*ClpP ^b^*
	Conserved ORFs	*ycf1*^c^ (×2)*, ycf2 ^c^* (×2)*, ycf3 ^b^, ycf4, ycf15 ^c,d^* (×2)

^a^ genes with one intron, ^b^ genes with two introns, ^c^ two gene copies in IRs, ^d^ genes only *M. paniculata* have.

**Table 2:** Summary statistics of chloroplast genomes of the *Meconopsis* species.

Genome Feature	*M. paniculata*	*M. pinnatifolia*	*M. racemosa*	*M. henrici*	*M. punicea*	*M. quintuplinervia*	*M. pseudohorridula*	*M. simplicifolia*	*M. betonicifolia*	*M. horridula*	*M. integrifolia*	*M. bella*
Genome size (bp)	152,887	153,557	153,763	153,788	153,281	154,997	154,190	152,772	151,935	153,785	151,864	153,073
LSC size (bp)	83.366	84,067	83,868	83,644	83,999	85,153	84,064	83,778	83,147	83,901	82,809	83,562
SSC size (bp)	17,857	17,894	17,905	17,822	17,728	17,876	17,770	17,646	17,746	17,898	17,753	17,833
IR size (bp)	25,832	25,798	25,995	26,161	25,777	25,984	26,178	25,674	25,521	25,993	25,649	25,839
Number of genes	133	131	129	133	133	133	134	131	131	127	127	133
Protein genes	88	86	84	88	88	88	88	84	86	87	88	88
tRNA genes	37	37	37	37	37	37	37	37	37	29	29	37
rRNA genes	8	8	6	8	8	8	8	8	8	8	8	8
Duplicated genes in IRs	19	18	17	19	19	19	20	19	18	19	19	19
GC content (%)	38.8%	38.8%	38.8%	38.5%	38.5%	38.5%	38.6%	38.7%	38.8%	38.8%	38.8%	38.9%
GC content in LSC (%)	37.3%	37.3%	37.3%	37.0%	37.0%	37.1%	37.0%	37.3%	37.3%	37.2%	37.4%	37.5%
GC content in SSC (%)	33.2%	33.3%	33.1%	32.8%	32.7%	32.8%	33.0%	33.0%	33.0%	33.2%	33.3%	33.5%
GC content in IRs (%)	43.1%	43.1%	43.1%	43.0%	42.9%	43.0%	43.0%	43.1%	43.1%	43.1%	43.1%	43.2%

## Data Availability

The chloroplast sequences of *M. pinnatifolia* and *M. paniculata* have been uploaded to GenBank and the accession numbers are OR521089 and OR521090.

## Supplementary Materials

The following supporting information can be downloaded at https://www.mdpi.com/article/10.3390/ijms25042193/s1.
