# Supplementary material for: Comparative Analysis of Chloroplast Genome of Meconopsis (Papaveraceae) Provides Insights into Their Genomic Evolution and Adaptation to High Elevation
Source: Int J Mol Sci. 2024 Feb 12;25(4):2193. doi: 10.3390/ijms25042193 (PMC10888623; doi:10.3390/ijms25042193)
Supplement: Supplementary file 1 [file ijms-25-02193-s001.zip › Figure S1/A.Meconopsis pinnatifolia.pdf]

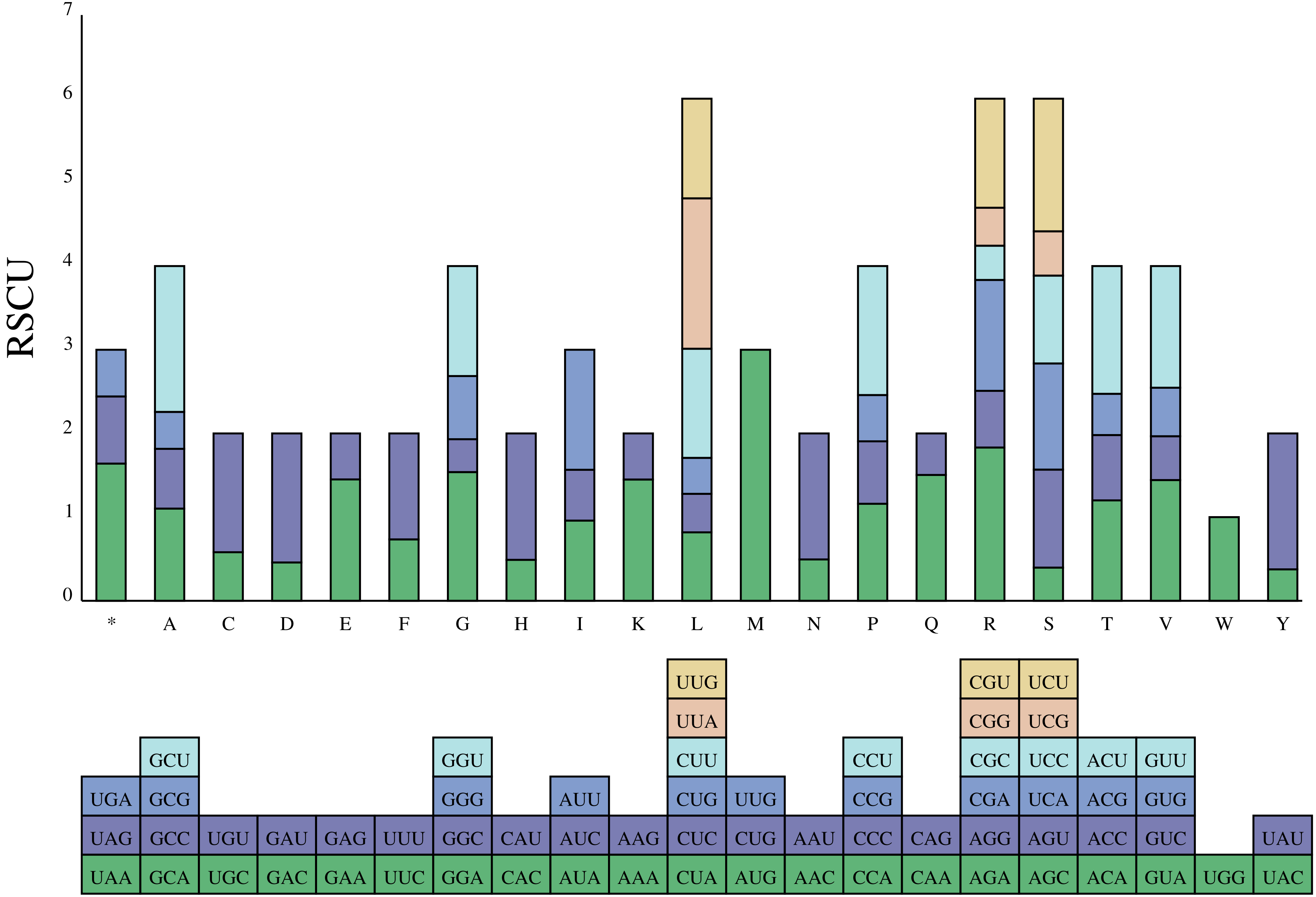
RSCU
0
1
2
3
4
5
6
7
*
A
C
D
E
F
G
H
I
K
L
M
N
P
Q
R
S
T
V
W
Y
UUG
CGU
UCU
UUA
CGG
UCG
GCU
GGU
CUU
CCU
CGC
UCC
ACU
GUU
UGA
GCG
GGG
AUU
CUG
UUG
CCG
CGA
UCA
ACG
GUG
UAG
GCC
UGU
GAU
GAG
UUU
GGC
CAU
AUC
AAG
CUC
CUG
AAU
CCC
CAG
AGG
AGU
ACC
GUC
UAU
UAA
GCA
UGC
GAC
GAA
UUC
GGA
CAC
AUA
AAA
CUA
AUG
AAC
CCA
CAA
AGA
AGC
ACA
GUA
UGG
UAC
