# Supplementary material for: Comparative Analysis of Chloroplast Genome of Meconopsis (Papaveraceae) Provides Insights into Their Genomic Evolution and Adaptation to High Elevation
Source: Int J Mol Sci. 2024 Feb 12;25(4):2193. doi: 10.3390/ijms25042193 (PMC10888623; doi:10.3390/ijms25042193)
Supplement: Supplementary file 1 [file ijms-25-02193-s001.zip › Figure 3.pdf]

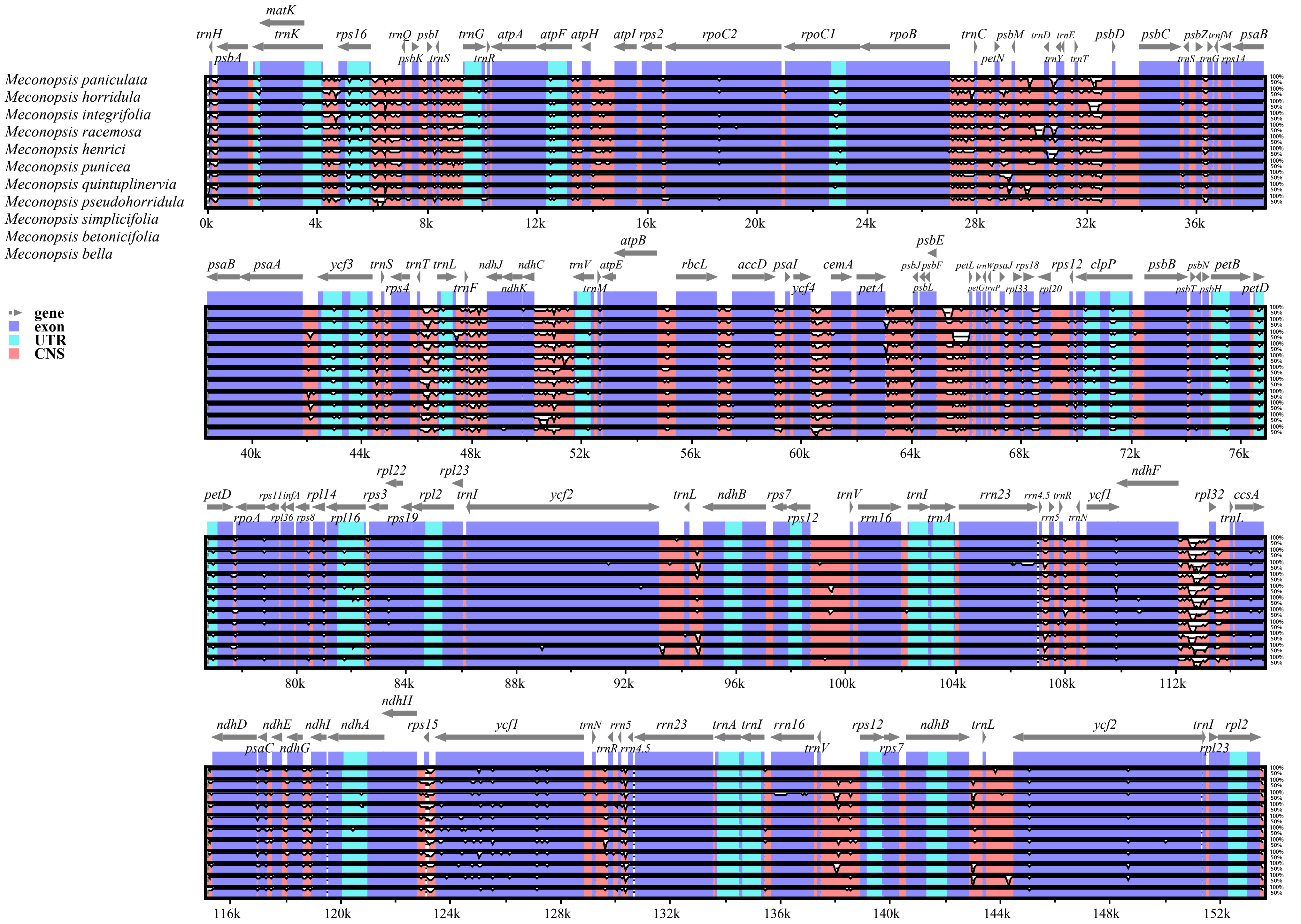

Meconopsis paniculata
Meconopsis horridula
Meconopsis integrifolia
Meconopsis racemosa
Meconopsis henrici
Meconopsis punicea
Meconopsis quintuplinervia
Meconopsis pseudohorridula
Meconopsis simplicifolia
Meconopsis betonicifolia
Meconopsis bella
gene
exon
UTR
CNS
matK
trnH
trnK
rps16
trnQ
psbI
trnG
atpA
atpF
atpH
atpI
rps2
rpoC2
rpoC1
rpoB
trnC
psbM
trnD
trnE
psbD
psbC
psbZ
trnfM
psaB
psbA
psbK
trnS
trnR
petN
trnY
trnT
trnS
trnG
rps14
0k
4k
8k
12k
16k
20k
24k
28k
32k
36k
atpB
psbE
psaB
psaA
ycf3
trnS
trnT
trnL
ndhJ
ndhC
trnV
atpE
rbcL
accD
psaI
cemA
psbJ
psbF
petL
trnW
psaJ
rps18
rps12
clpP
psbB
psbN
petB
rps4
trnF
ndhK
trnM
ycf4
petA
psbL
petG
trnP
rpl33
rpl20
psbT
psbH
petD
40k
44k
48k
52k
56k
60k
64k
68k
72k
76k
rpl22
rpl23
ndhF
petD
rps11
infA
rpl14
rps3
rpl2
trnI
ycf2
trnL
ndhB
rps7
trnV
trnI
rrn23
rrn4.5
trnR
ycf1
rpl32
ccsA
rpoA
rpl36
rps8
rpl16
rps19
rps12
rrn16
trnA
rrn5
trnN
trnL
80k
84k
88k
92k
96k
100k
104k
108k
112k
ndhH
ndhD
ndhE
ndhI
ndhA
rps15
ycf1
trnN
rrn5
rrn23
trnA
trnI
rrn16
rps12
ndhB
trnL
ycf2
trnI
rpl2
psaC
ndhG
trnR
rrn4.5
trnV
rps7
rpl23
116k
120k
124k
128k
132k
136k
140k
144k
148k
152k
